# Embedding Dimension Selection for Adaptive Singular Spectrum Analysis of EEG Signal

**DOI:** 10.3390/s18030697

**Published:** 2018-02-26

**Authors:** Shanzhi Xu, Hai Hu, Linhong Ji, Peng Wang

**Affiliations:** 1 State Key Laboratory of Precision Measurement Technology and Instruments, Department of Precision Instrument, Tsinghua University, Beijing 100084, China; xsz2016@mail.tsinghua.edu.cn (S.X.); huhai@mail.tsinghua.edu.cn (H.H.); 2 Division of Intelligent and Bio-mimetic Machinery, The State Key Laboratory of Tribology, Tsinghua University, Beijing 100084, China; jilh@mail.tsinghua.edu.cn

**Keywords:** EEG signal, rhythm extraction, adaptive singular spectrum analysis, embedding dimension selection

## Abstract

The recorded electroencephalography (EEG) signal is often contaminated with different kinds of artifacts and noise. Singular spectrum analysis (SSA) is a powerful tool for extracting the brain rhythm from a noisy EEG signal. By analyzing the frequency characteristics of the reconstructed component (RC) and the change rate in the trace of the Toeplitz matrix, it is demonstrated that the embedding dimension is related to the frequency bandwidth of each reconstructed component, in consistence with the component mixing in the singular value decomposition step. A method for selecting the embedding dimension is thereby proposed and verified by simulated EEG signal based on the Markov Process Amplitude (MPA) EEG Model. Real EEG signal is also collected from the experimental subjects under both eyes-open and eyes-closed conditions. The experimental results show that based on the embedding dimension selection method, the alpha rhythm can be extracted from the real EEG signal by the adaptive SSA, which can be effectively utilized to distinguish between the eyes-open and eyes-closed states.

## 1. Introduction

Electroencephalography (EEG) recordings from the scalp reflect the electrophysiological activity of the brain neurons. The features of the spontaneous EEG signal contain different physiological and pathological information [1]. For example, alpha rhythm (*α*-rhythm) reflects attentional demands and beta rhythm (*β*-rhythm) reflects emotional and cognitive processes [2]. Theta rhythm (*θ*-rhythm) is related to moral actions [3], while delta rhythm (*δ*-rhythm) is an indicator of attention to internal processing during performance of mental tasks [4]. Due to the advantage of abundant information in the brain, EEG has been widely studied in the area of physiological status monitoring, intelligent rehabilitation therapy and brain computer interface (BCI) system [5,6,7].

Rhythm extraction is very important for the research and application of EEG signal. However, the recorded EEG signal usually contains large amounts of artifacts, such as electrooculogram (EOG), electromyography (EMG), electrocardiography (ECG), baseline drift and so on, in consistence with common interference and random noise originating from measurement system. Especially, the artifacts show considerably larger amplitude than the spontaneous EEG signal, which lead to an unsatisfied low signal-to-noise ratio (SNR) [8]. In addition, the frequency spectrum of artifacts overlaps with that of the spontaneous EEG signal. Therefore, traditional methods based on frequency spectrum analysis, like Fourier Transform, are difficult to accomplish the artifacts removal and rhythms extraction. What’s more, the brain rhythm depends on the experimental subject to a large extent and the artifacts vary significantly with the measurement environment. Consequently, the rhythms extraction from the EEG signal has attracted much attention in recent studies.

To extract the desired rhythms accurately from the contaminated EEG signal, various processing methods have been proposed for artifacts removal [9,10,11]. In multi-channel EEG recordings, a conventional approach of visual inspection is useful for artifacts removal. However, this method is time consuming, laborious and unrealizable for single-channel EEG recording. Therefore, many efforts have been paid on automated detection and correction for artifacts removal. He et al. combined regression analysis (RA) and adaptive filtering (AF) for removal of ocular artifacts [12]. This method required a separately recorded ocular artifacts signal as a reference, which increased the complexity of the measurement system. Jadhav et al. proposed an automated detection and correction method of eye blink and muscular artifacts based on fast independent component analysis (FastICA) and discrete waveform transform (DWT) [13]. Then this method is further improved and realized in a low-complexity and reliable system without the need of any reference electrode [14]. Machine learning techniques were recently introduced to the processing of EEG signals. For instance, Chai et al. used an autoregressive (AR) model and sparse-deep belief networks (sparse-DBN) combined with independent component analysis (ICA) to remove artifacts and discriminateEEG-based driver fatigue, which obtained satisfied classification performance between fatigue and alert states [15]. However, this method required abundant labeled EEG samples for training the parametric model and suffered from the cost of high computational complexity, which is not applicable for portable or wearable devices.

Singular spectrum analysis (SSA) is a model-free method developed from Karhunen-Loeve decomposition theory [16]. SSA works well for single channel signals as well as multichannel signals. So, it allows the exploitation of SSA for a variety of applications including biomedical signal processing such as signal restoration, change detection, segmentation, anomaly detection and prediction [17]. In terms of biomedical signal, the recorded biomedical information is often a combination of a number of source signals, artifact and noise, which possesses nonlinear and non-stationary characteristics [18]. SSA is utilized to find the structure of nonlinear and non-stationary signal and enables the separation of different sources even overlapping in the time-frequency space. In recent years, SSA has been successfully applied in the research of artifacts removal and rhythm extraction from the EEG signal. For instance, the research group of Maddirala [19] analyzed single channel EEG signal by SSA processing and successfully realized the motion artifact removal. Then the EOG artifact was furtherly removed by means of combined singular spectrum analysis and adaptive noise canceler [20]. SSA was applied by the Mohammadi group [21,22] to automatically extract the sleep EEG rhythm and successfully discriminated between different sleep states. Akar et al. proposed an approach based on wavelet and SSA to eliminate noise and extract desired components from the EEG signal, which was then effectively applied to the analysis of brain dynamics [23].

During the SSA processing of EEG signal, embedding dimension selection and grouping rule are two critical issues. For the issue of grouping rule, various methods have been proposed and achieved good performance in different applications. The conventional grouping rule was performed according to the magnitudes of eigenvalues, which was related with the power of each RC [24]. Then a new grouping rule based on the local mobility of the eigenvectors was proposed to remove the motion artifact, which performed better than the traditional method [19]. Based on the similarity of the eigenvalues and the peak frequency of RC, Hai et al. proposed another efficient grouping rule enabling SSA to be adaptive to EEG signals containing different levels of artifacts and rhythms [25]. However, for the issue of embedding dimension selection, to the best of our knowledge, there is no explicit rule for embedding dimension selection. On one hand, a general strategy is that the embedding dimension L should capture at least one period of the lowest frequency component of interest, i.e., $L>f_{s}/f_{r}$, where $f_{s}$ is the sampling rate and $f_{r}$ is the minimum frequency [26,27]. One the other hand, to get a satisfied result, the embedding dimension L should be chosen sufficiently large enough so that L-lagged vector incorporates an essential part of the EEG features [28]. The larger the embedding dimension is, the more detailed is the decomposition of the EEG signal. The most detailed decomposition is achieved when the embedding dimension is approximately equal to half of the EEG signal length. However, a larger L corresponds to an increased amount of computation and time complexity. In addition, for the EEG signal with complex structures, too large L may produce component mixing with each other [29], which lead to undesirable decomposition of brain rhythm of interest.

An embedding dimension selection method of the adaptive SSA for EEG signal processing is proposed in this paper. Based on the embedding dimension selection method, the frequency bandwidth of each reconstructed component is limited to a particular band of the brain rhythm of interest. At the same time, this method avoids component mixing in the SVD step. The experimental results show that, with the aid of the proposed embedding dimension selection method, the adaptive SSA performs successfully in the alpha rhythm extraction and distinguishing between eyes-open and eyes-closed states.

## 2. Adaptive Singular Spectrum Analysis Method for EEG Processing

Regarding the recorded one-dimensional time series of EEG signal **s** = (s_1_,s_2_,…,s_N_)^T^, with the superscript T denoting the transpose operator, SSA consists of two complementary stages: decomposition and reconstruction. Each stage includes two separate steps.

The decomposition stage includes time-delay embedding operation followed by singular value decomposition (SVD). In the time-delay embedding step, one-dimensional time series **s** is mapped into the multi-dimensional space to construct the trajectory matrix

(1) $$X=\left(s_{1},s_{2},\ldots ,s_{L}\right)=\left[\begin{array}{cccc} s_{1} & s_{2} & \cdots & s_{L}\\ s_{2} & s_{3} & \cdots & s_{L+1}\\ \vdots & \vdots & \ddots & \vdots \\ s_{K} & s_{K+1} & \cdots & s_{N} \end{array}\right]$$

where L denotes the embedding dimension and K (K = N − L + 1) denotes the indices of the L-dimensional time-delay vectors. In the SVD step, the trajectory matrix is processed by SVD and decomposed into a series of rank-one elementary matrices

(2) $$X=X_{1}+X_{2}+\ldots +X_{r}=\overset{r}{\underset{i=1}{\sum }}\sqrt{\lambda_{i}}u_{i}{v_{i}}^{T}$$

where $\lambda_{i}$ are the eigenvalues of the covariance matrix $X^{T}X$ in decreasing order of magnitude ($\lambda_{1}$ ≥ $\lambda_{2}$ ≥ … ≥ $\lambda_{r}$ > 0). The left singular vectors $u_{i}$ are the eigenvectors of the covariance matrix $XX^{T}$ and the right singular vectors $v_{i}$ are the eigenvectors of the covariance matrix $X^{T}X$. Therefore, the trajectory matrix X is projected into the orthogonal space spanned by the left and right singular vectors. Each principal component subspace contains different characteristics of the EEG signal.

The reconstruction stage includes grouping and diagonal averaging. In the grouping step, the reconstructed components containing the same characteristics are grouped to indicate different brain rhythms, artifacts or noise. Let A = (c_1_,c_2_,…,c_m_) (m < r) be the corresponding grouped sequences. Then, the reconstructed matrix is

(3) $$X_{c}=\overset{c_{m}}{\underset{i=c_{1}}{\sum }}X_{i}=\left[\begin{array}{cccc} {\bar{s}}_{1}^{c} & {\bar{s}}_{2}^{c} & \cdots & {\bar{s}}_{L}^{c}\\ {\bar{s}}_{2}^{c} & {\bar{s}}_{3}^{c} & \cdots & {\bar{s}}_{L+1}^{c}\\ \vdots & \vdots & \ddots & \vdots \\ {\bar{s}}_{K}^{c} & {\bar{s}}_{K+1}^{c} & \cdots & {\bar{s}}_{N}^{c} \end{array}\right]$$

Finally, $X_{c}$ is transformed into a new one-dimensional time series $s_{c}={\left(s_{1}^{c},s_{2}^{c},\ldots ,s_{N}^{c}\right)}^{T}$ in the diagonal averaging step, which represents the particular characteristic of the EEG signal.

Thus, as can be seen that the embedding dimension and grouping sequence selection are the two critical parameters of the SSA process. According to the magnitude of the singular value and the dominant frequency of each reconstructed component, an adaptive grouping rule has been proposed to solve the problem of grouping sequence selection [25]. In the following section, an embedding dimension selection method of adaptive SSA is presented for the purpose of rhythm extraction from the EEG signal.

In the above SVD step, the singular values of trajectory X are tied up with the eigenvalues of the covariance matrix $X^{T}X$ via the relation $\sigma =\sqrt{\lambda }$. The covariance matrix is represented by

(4) $$X^{T}X=\left[\begin{array}{cccc} s_{1}^{T}s_{1} & s_{1}^{T}s_{2} & \cdots & s_{1}^{T}s_{L}\\ s_{2}^{T}s_{1} & s_{2}^{T}s_{2} & \cdots & s_{2}^{T}s_{L}\\ \vdots & \vdots & \ddots & \vdots \\ s_{L}^{T}s_{1} & s_{L}^{T}s_{2} & \cdots & s_{L}^{T}s_{L} \end{array}\right]$$

It is a symmetric positive semidefinite matrix. Generally, it satisfies the conditions N≫L and K≫L during the application of EEG signal processing. Thus, the principal diagonal elements and the elements parallel to the principal diagonal are approximately equal, namely

(5) $$z_{l}=\sum_{n=1}^{K}s_{n}s_{n-l}≃s_{L-m}s_{L-l-m}(L=0,1,\ldots ,L-1,m=0,1,\ldots ,L-1-1)$$

where $z_{l}$ is the auto covariance with delayed points l. Then covariance matrix $X^{T}X$ can be rewritten as

(6) $$T_{K,L}=X^{T}X=\left[\begin{array}{cccc} z_{0} & z_{1} & \cdots & z_{L-1}\\ z_{1} & z_{0} & \cdots & z_{L-2}\\ \vdots & \vdots & \ddots & \vdots \\ z_{L-1} & z_{L-2} & \cdots & z_{0} \end{array}\right]$$

Based on the theorem of the symmetric Toeplitz matrix [30,31], the eigenvalues of $T_{K,L}$ are calculated as

(7) $$\lambda_{p}=\overset{L-1}{\underset{j=-L+1}{\sum }}z_{\left|j\right|}e^{i2\pi \mathrm{jp}/L}$$

To analyze the relationship between the eigenvalues of the covariance matrix and the frequency spectrum of the EEG signal, the circular operator is introduced here to represent

(8) $$z_{l}≃s_{K}^{T}P_{K}^{l}s_{K}=s_{K}^{T}P_{K}^{-l}s_{K}$$

where $s_{K}$ denotes the first K elements of s and $P_{K}$ denotes the basic circulant matrix. Hence, $P_{K}$ is utilized to construct the relationship between the eigenvalues of $T_{K,L}$ and the discrete Fourier transform (DFT) of $s_{K}$. The K×K Fourier matrix is represented as

(9) $$F_{K}=\frac{1}{\sqrt{K}}\left[\begin{array}{cccc} 1 & 1 & \cdots & 1\\ 1 & \omega_{K}^{1} & \cdots & \omega_{K}^{K-1}\\ \vdots & \vdots & \ddots & \vdots \\ 1 & \omega_{K}^{K-1} & \cdots & \omega_{K}^{\left(K-1\right)\left(K-1\right)} \end{array}\right]$$

where $\omega_{K}=e^{i2\pi /K}$ and $F_{K}$ is unitary matrix. With the aid of the matrix $F_{K}$, the basic circulant matrix can be rewritten as [32]

(10) $$P_{K}=F_{K}\Lambda_{K}F_{K}^{H}=F_{K}\left[\begin{array}{cccc} 1 & 0 & \cdots & 0\\ 0 & \omega_{K} & \cdots & 0\\ \vdots & \vdots & \ddots & \vdots \\ 0 & 0 & \cdots & \omega_{K}^{\left(K-1\right)} \end{array}\right]F_{K}^{H}$$

where $F_{K}^{H}$ is the conjugate transpose of the matrix $F_{K}$. In addition, the form of the inverse DFT is

(11) $$s_{K}=F_{K}^{H}{\hat{s}}_{K}=F_{K}^{H}{\left({\hat{s}}_{0},{\hat{s}}_{1},\ldots ,{\hat{s}}_{K-1}\right)}^{T}$$

where ${\hat{s}}_{K}$ denotes the frequency spectrum of the time series $s_{K}$. Upon substituting Equations (10) and (11) into Equation (8), we get

(12) $$z_{l}≃{\hat{s}}_{K}^{T}\Lambda_{K}^{l}{\hat{s}}_{K}={\hat{s}}_{K}^{T}\Lambda_{K}^{-l}{\hat{s}}_{K}$$

Based on Equations (7) and (12), the eigenvalues are written in the form

(13) $$\lambda_{p}={\hat{s}}_{K}^{T}\left(\overset{L-1}{\underset{j=-L+1}{\sum }}\Lambda_{K}^{j}e^{i2\pi \mathrm{jp}/L}\right){\hat{s}}_{K}=\overset{K-1}{\underset{q=0}{\sum }}{\left|{\hat{s}}_{q}\right|}^{2}\zeta_{p,q}$$

where the coefficients are given by

(14) $$\zeta_{p,q}=\overset{L-1}{\underset{j=-L+1}{\sum }}e^{i2\pi j\left(p/L+q/K\right)}=1+2\overset{L-1}{\underset{m=1}{\sum }}\cos2\pi m\left(p/L+q/K\right)$$

**Figure 1:**
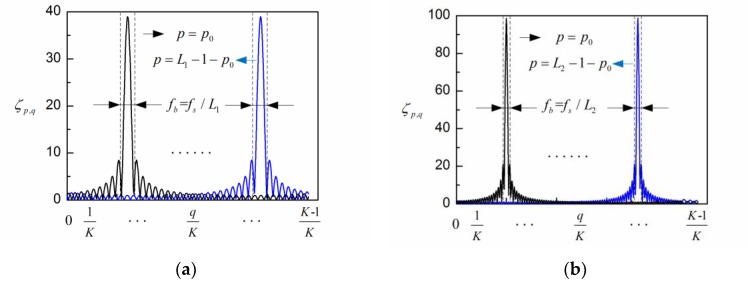
The coefficients $\zeta_{p,q}$ for different parameters of L and p: (**a**) $L_{1}$ = 20; (**b**) $L_{2}$ = 50.

It can be seen that the eigenvalues of $T_{K,L}$ are determined by the magnitude ${\left|{\hat{s}}_{q}\right|}^{2}$ and coefficients $\zeta_{p,q}$. The coefficients $\zeta_{p,q}$ for different parameters are shown in Figure 1. The coefficient at −p×K/L is the dominant component, while the coefficients between −p×K/L−0.5×K/L and −p×K/L+0.5×K/L are the major parts of all the components. Based on Equation (13), each eigenvalue of the normalized covariance matrix $T_{K,L}$ can be approximated by the mean value of a portion of the power spectrum of $s_{K}$, whose width is roughly K/L [33]. For the recorded EEG signal at a sampling rate $f_{s}$, the frequency resolution of the DFT is $f_{s}/K$. Therefore, the frequency bandwidth of each reconstructed component can be expressed by

(15) $$f_{b}=K/L\times f_{s}/K=f_{s}/L$$

As a consequence, the frequency bandwidth $f_{b}$ of each reconstructed component is limited to $f_{s}/L$. In order to remove the frequency components outside the $f_{b}$, with $f_{b}$ denoting the bandwidth of the brain rhythm of interest, the embedding dimension L should satisfy the condition $L\geq f_{s}/f_{b}$. Otherwise, the reconstructed components in the grouping step will contain frequency components outside the bandwidth of the brain rhythm of interest.

The analysis presented above shows that the frequency bandwidth of each reconstructed component is determined by the embedding dimension. Besides, the rate of change in the trace of Toeplitz matrix $T_{K,L}$ is associated with the embedding dimension [34] via the relation

(16) $${\mathrm{Tr}}^{L,N}-{\mathrm{Tr}}^{L-1,N}=\overset{K}{\underset{j=L}{\sum }}s_{j}^{2}$$

where the trace of $T_{K,L}$ is defined as

(17) $${\mathrm{Tr}}^{L,N}=\mathrm{tr}\left(T_{K,L}\right)=\overset{L}{\underset{j=1}{\sum }}\lambda_{j}^{L,N}$$

It can be seen that the change rate of the trace is large for small embedding dimensions and decreases to the attain a minimum value at L = K. It means that the increase of information for all the frequency components slows down gradually when the embedding dimension L increases. Therefore, a smaller value of L should be chosen when the change rate of the trace is small. Otherwise, for nonlinear and nonstationary EEG signal with complicated structures, too large L can lead to component mixing with each other, which result in unsatisfied brain rhythm extraction.

The recorded EEG signal contains different brain rhythms, artifacts and noise. In particular, the frequency spectrum of the brain rhythms overlaps with that of the artifacts and noise. Therefore, in order to avoid component mixing in the rhythm extraction from the EEG signal by the adaptive SSA, the embedding dimension is selected according to the rule $L=f_{s}/f_{b}$, where $f_{b}$ is determined by the frequency bandwidth of the brain rhythm to be extracted.

## 3. Simulation Results and Discussion

### 3.1. Markov Process Amplitude EEG Model

The simulated EEG signal consists of three parts: the spontaneous EEG signal, the artifacts and the measurement noise. The spontaneous EEG signal possesses the characteristic rhythmic oscillations and stochastic processes. The artifacts are represented by the EOG and baseline drift. The measurement noise is simulated by the Gaussian white noise.

The spontaneous EEG signal is generated based on the Markov Process Amplitude (MAP) EEG model [35,36]. The rhythmic oscillation is represented by the sinusoidal wave, while the stochastic process is represented by the first-order Markov process. Therefore, the spontaneous EEG is produced by the sinusoidal wave modulated by the first-order Markov process. Considering that the spontaneous EEG consists of several oscillations, the MPA EEG model is given by

(18) $$\{\begin{array}{c} s\left(n\Delta t\right)=\overset{K}{\underset{i=1}{\sum }}a_{i}\left(n\Delta t\right)\sin\left(2{\pi f}_{i}n\Delta t+\theta_{i}\right)+v_{\alpha }\left(n\Delta t\right)+v_{n}\left(n\Delta t\right)\\ a_{i}\left(\left(n+1\right)\Delta t\right)=\gamma_{i}a_{i}\left(n\Delta t\right)+\xi_{i}\left(n\Delta t\right),\;\;\;\;\;\;\;\;\;\;\;\;\;\;\;\;\;\;\;\;\;\;\;\;\;0<\gamma_{i}<1 \end{array}$$

where K is the number of rhythms, n is the number of sampling points, Δt is the sampling interval, f is the oscillation frequency, θ is the initial phase, while $v_{\alpha }$ and $v_{n}$ represent the artifacts and Gaussian white noise, respectively.

Equation (18) shows that the amplitude of the first-order Markov process at the succeeding time (n+1)Δt depends only on the amplitude at the current time Δt and is independent of the amplitudes of any other time. ξ represents the stochastic feature of amplitudes between the two steps of the process, for which the variance of ξ is $\sigma^{\xi }$ and the mean of ξ is zero. There are two critical parameters in the first-order Markov process: γ and ξ. γ denotes the correlation of the amplitudes between adjacent steps and $\sigma^{\xi }$ denotes the strength of the stochastic process.

**Figure 2:**
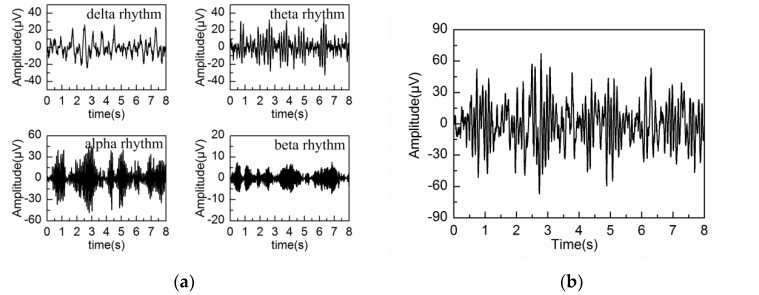
The simulation process of the spontaneous electroencephalography (EEG) signal: (**a**) simulated delta, theta, alpha and beta rhythm; (**b**) the spontaneous EEG signal constructed by the superposition of the four rhythms.

The procedure for the simulation of the spontaneous EEG signal based on MPA EEG model is given in Figure 2. Firstly, by means of the maximum likelihood method, the parameters of γ and ξ are estimated from the power spectrum density (PSD) of the real EEG signal. Then, four brain rhythms are generated using the above estimated parameters, as shown in Figure 2a. The four brain rhythms are delta rhythm (1−4 Hz), theta rhythm (4−8 Hz), alpha rhythm (8−13 Hz) and beta rhythm (13−30 Hz), whose oscillation frequencies are set at the middle points of the corresponding frequency ranges. Finally, the spontaneous EEG is constructed as a superposition of the four brain rhythms, as shown in Figure 2b. In the simulation, the sampling frequency is 200 Hz and the sampling time is 8s. All of the simulation parameters are shown in Table 1.

### 3.2. Adaptive Singular Spectrum Analysis for Simulated EEG signal

**Figure 3:**
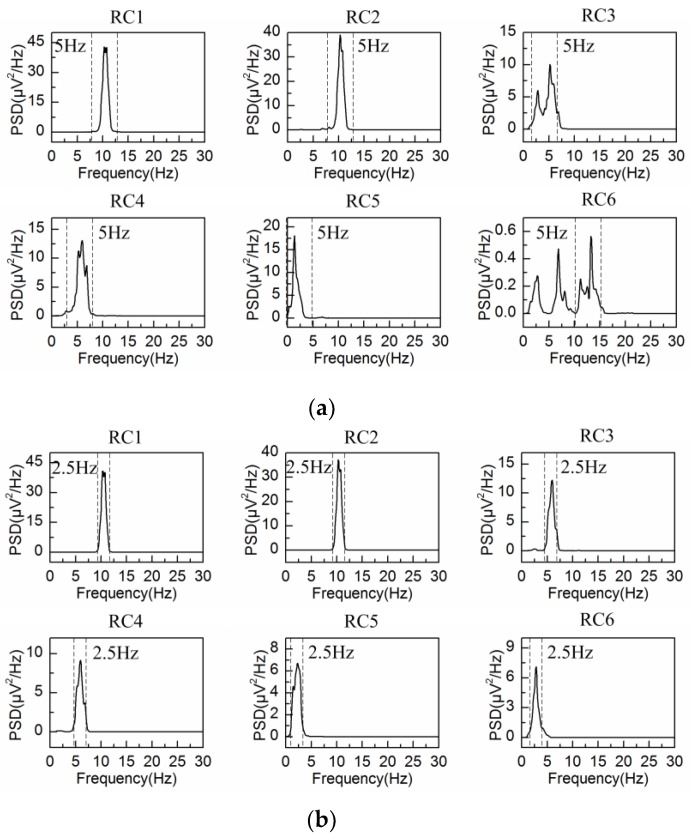
Power spectrum density (PSD) of the first six reconstructed components for the spontaneous EEG signal: (**a**) L = 40; (**b**) L = 80.

The spontaneous EEG signal is processed by the adaptive SSA and the embedding dimension is set at L = 40 ($f_{s}/f_{b\left(\alpha \right)}$) and L = 80 ($2f_{s}/f_{b\left(\alpha \right)}$), with $f_{b\left(\alpha \right)}$ = 5 Hz denoting the bandwidth of alpha rhythm. The PSD of the first six reconstructed components are shown in Figure 3a,b, respectively. In agreement with the results from Figure 1, the bandwidth of each reconstructed component is limited to $f_{s}/L$ (between two dashed lines in Figure 3), i.e., 5 Hz for L = 40 and 2.5 Hz for L = 80. It is observed from Figure 3 that RC1 and RC2 reflect the frequency feature of the alpha rhythm in both situations. Then the alpha rhythm is reconstructed according to the adaptive grouping rule [25].

For the purpose of evaluating the reconstruction performance of the alpha rhythm, an error parameter is defined as

(19) $$\varepsilon_{\mathrm{ave}}=\frac{1}{N}\overset{N}{\underset{i=1}{\sum }}\left|p_{\alpha }\left(i\right)-p_{r}\left(i\right)\right|$$

where $\varepsilon_{\mathrm{ave}}$ is the average error of the PSD between the simulated alpha rhythm and the reconstructed alpha rhythm, $p_{\alpha }$ is the PSD of the simulated alpha rhythm, $p_{r}$ is the PSD of the reconstructed alpha rhythm and N is the length of the PSD.

**Figure 4:**
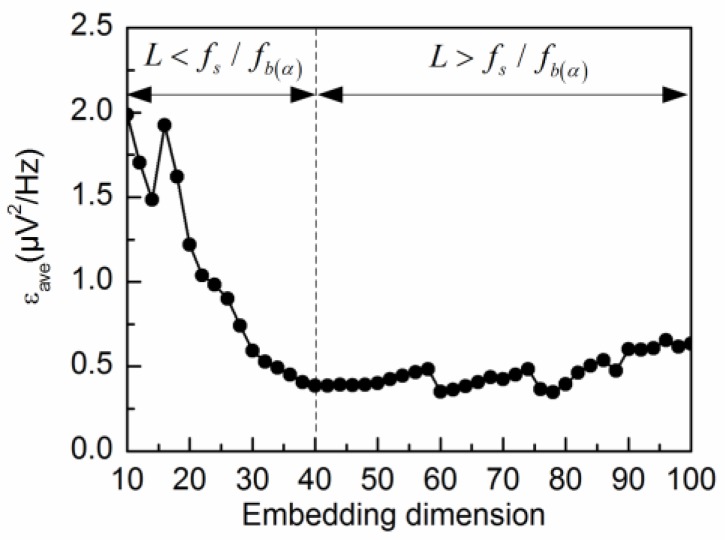
$\varepsilon_{\mathrm{ave}}$ corresponding to different embedding dimensions L during the alpha rhythm extraction from the spontaneous EEG signal.

The alpha rhythm is extracted from the spontaneous EEG signal by different values of L. The calculated errors $\varepsilon_{\mathrm{ave}}$ are shown in Figure 4. As shown in the figure, $\varepsilon_{\mathrm{ave}}$ decreases gradually for small values of L (L < 40), while $\varepsilon_{\mathrm{ave}}$ reaches a relatively steady state for large values of L (L > 40). In accordance with the theoretical analysis, the alpha rhythm can be effectively extracted from the spontaneous EEG signal by the adaptive SSA when the embedding dimension is set at $L=f_{s}/f_{b\left(\alpha \right)}$. Therefore, $\varepsilon_{\mathrm{ave}}$ attains a minimum value at L = 40.

In order to further analyze the effect of the embedding dimension on the performance of the alpha rhythm extraction, the artifacts and noise are superimposed on the spontaneous EEG signal to construct the simulated EEG signal. The artifacts consist of two parts: EOG and baseline drift. EOG is represented by the triangular waveform with low frequency and high amplitude, which is the main source of artifacts and is caused by eye blinks and ocular movement. The baseline drift is characterized by low-frequency sinusoidal wave, which originates from head or body movement. In addition, Gaussian white noise is used to simulate the measurement noise. The simulation parameters of the artifacts and noise are shown in Table 1.

**Figure 5:**
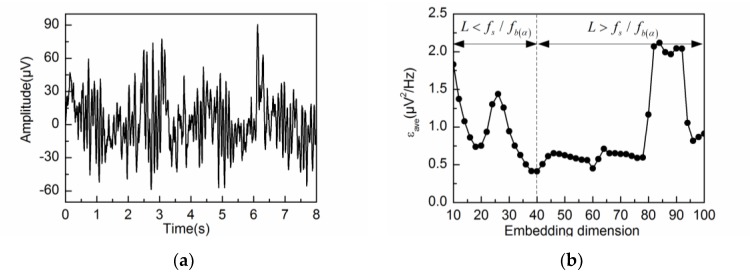
Extracting the alpha rhythm from the simulated EEG signal by different embedding dimensions L: (**a**) the simulated EEG signal; (**b**) $\varepsilon_{\mathrm{ave}}$ corresponding to different embedding dimensions L.

**Figure 6:**
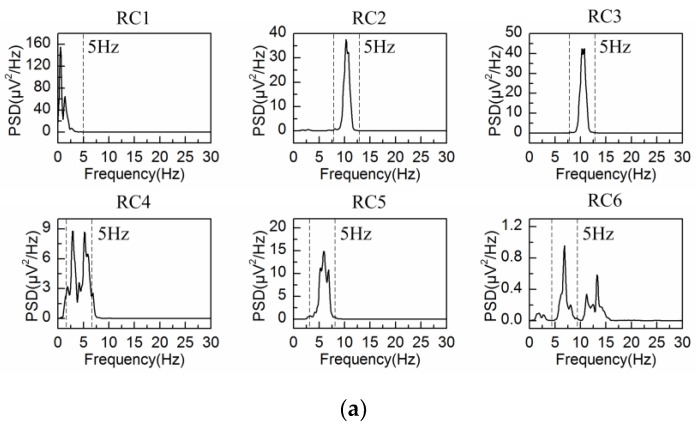

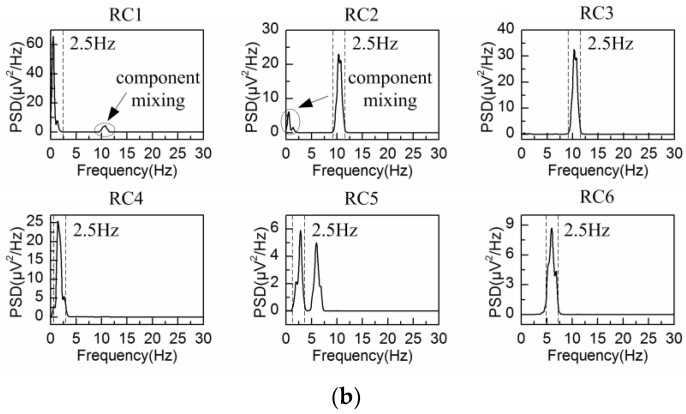
PSD of the first six reconstructed components for the simulated EEG signal: (**a**) L = 40; (**b**) L = 80.

Figure 5a shows the simulated EEG signal with artifacts and noise. The average error $\varepsilon_{\mathrm{ave}}$ corresponding to the embedding dimension L is shown in Figure 5b. It can be seen from Figure 5b that $\varepsilon_{\mathrm{ave}}$ decreases gradually for small values of L (L < 40) and attains a minimum value at L = 40. However, for large values of L (L > 40), $\varepsilon_{\mathrm{ave}}$ shows a general increasing trend with significant fluctuations. In particular, $\varepsilon_{\mathrm{ave}}$ becomes much worse when L > 80. Figure 6a,b illustrate the PSD of the first six reconstructed components by L = 40 and 80. As can be observed in Figure 6, RC2 and RC3 reflect the frequency feature of the alpha rhythm. However, RC1 and RC2 suffer from component mixing for the alpha rhythm extraction when using L = 80. These two reconstructed components contain an admixture of frequency features from both alpha rhythm and artifacts.

**Figure 7:**
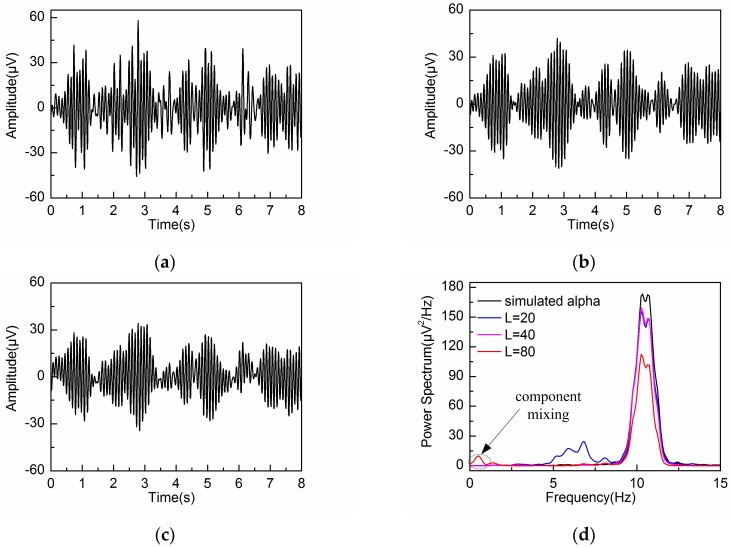
Extracted alpha rhythm: (**a**) L = 20; (**b**) L = 40; (**c**) L = 80; (**d**) PSD of the extracted alpha rhythm by different embedding dimensions L, as compared with that of the simulated alpha rhythm.

The extracted alpha rhythms by embedding dimensions L = 20, 40 and 80 are illustrated in Figure 7a–c, respectively. Figure 7d shows the PSD of the extracted alpha rhythm for the three different embedding dimensions, compared with that of the simulated alpha rhythm. It can be seen that the alpha rhythm can be extracted effectively by L = 20 and 40. Due to the effects of artifacts and noise, the PSD of the extracted alpha rhythm by L = 20 and 40 are lower than that of the simulated alpha rhythm. When the embedding dimension is set at L = 20, the extracted waveform contains frequency components outside the alpha band because of the wide frequency bandwidth of the reconstructed components. When the embedding dimension is set at L = 80, the decomposition step leads to component mixing. Some of the frequency components of the alpha rhythm are mixed in the other reconstructed components. Therefore, the PSD of the extracted alpha rhythm is much lower than that of the simulated alpha rhythm. In addition, the PSD contains frequency feature from the artifacts due to the same reason of component mixing. So it is concluded that the adaptive SSA results in the best alpha rhythm extraction when the embedding dimension is set at L = 40.

## 4. Experimental Results and Discussion

### 4.1. Measurement Setup and Experimental Procedure

The real EEG signal was obtained by means of the MP36 data acquisition and analysis system (BIOPAC Systems Inc., Goleta, CA, USA). In order to improve the common mode rejection ratio (CMRR) of the measurement setup, three electrodes system was used to conduct the experiment. The Ag/AgCl electrode was adopted as the recording electrode, which was flushed with conductive gel and then attached to the frontal region of the subject’s scalp. The other two electrodes serving as a ground and a reference were attached to the earlobe and mastoid, respectively.

Three male and three female subjects aged 20 to 25 years participated in the experiment. All of the subjects were asked to refrain from psychoactive substances for at least 4 h prior to the experiments. The experimental procedures were as follows. Firstly, the subject relaxed with eyes closed for 10 min. Next, the subject opened eyes and focused on the cross symbol displayed on the computer screen. Finally, the subject kept eyes open for 60 s followed by a period with eyes closed for 60 s, and repeated this procedure 5 times. In the experiment, the real EEG signal is recorded at the sampling rate of 200 Hz. To obtain the desired segments of eyes-open and eyes-closed states, the segments lasting for 8 s were cut off from the middle of each period. Consequently, 30 segments of eyes-open state and 30 segments of eyes-closed state were obtained in total.

### 4.2. Adaptive Singular Spectrum Analysis for Real EEG Signal

**Figure 8:**
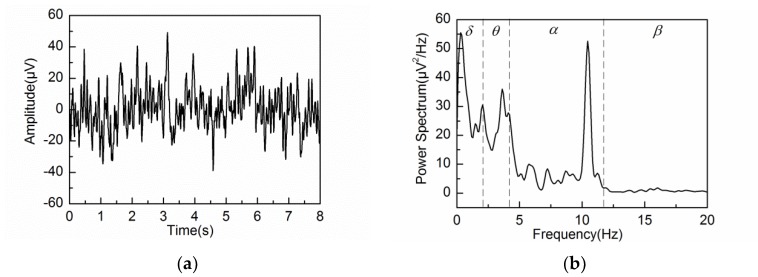
The real EEG signal and corresponding PSD under eyes-closed condition: (**a**) the real EEG signal under eyes-closed condition; (**b**) PSD of the real EEG signal under eyes-closed condition.

**Figure 9:**
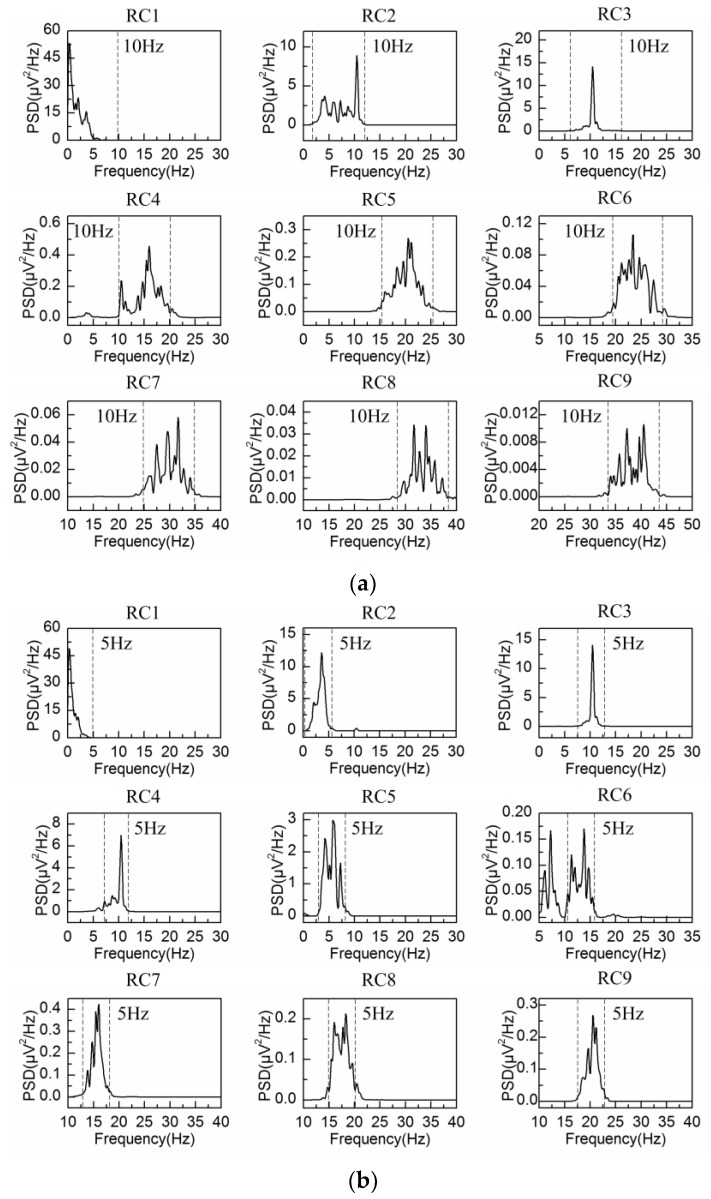

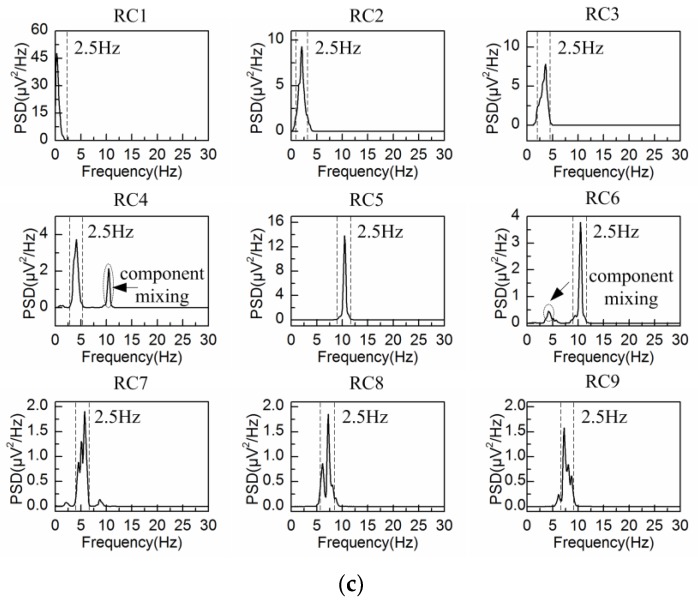
PSD of the first nine reconstructed components of the real EEG signal: (**a**) L = 20; (**b**) L = 40; (**c**) L = 80.

Figure 8a,b show the obtained EEG signal and the corresponding PSD under eyes-closed condition. The PSD of the first nine reconstructed components by L = 20, 40 and 80 are illustrated in Figure 9a−c, respectively. In agreement with the simulation results, the PSD of each reconstructed component is constrained within the frequency bandwidth of $f_{s}/L$, i.e., 10 Hz for L = 20, 5 Hz for L = 40 and 2.5 Hz for L = 80.

When the embedding dimension is set at L = 20, the PSD of each reconstructed component is limited to the frequency bandwidth of 10 Hz. For example, the bandwidth of RC2 is from 2 Hz to 12 Hz. Because RC2 contains the frequency features from delta, theta and alpha rhythms, it is hard to extract the alpha rhythm effectively. When the embedding dimension is set at L = 40, the PSD of each reconstructed component is limited to the frequency bandwidth of 5 Hz, which represents the frequency characteristic of alpha band well. Therefore, RC3 and RC4 effectively reflect the frequency feature of alpha rhythm. In particular, the frequency range of RC6 is from 5 Hz to 15 Hz. However, the peak value of PSD is only 0.17 ${\mu V}^{2}/\mathrm{Hz}$. So, PC6 mainly reflects the feature of the noise. When the embedding dimension is set at L = 80, RC4 and RC6 suffer from the problem of component mixing. The PSD of RC4 at the frequencies of 4.1 Hz and 10.5 Hz are 3.73 and 2.13 ${\mu V}^{2}/\mathrm{Hz}$, while The PSD of PC6 at the same frequencies of 4.1 Hz and 10.5 Hz are 0.42 and 3.77 ${\mu V}^{2}/\mathrm{Hz}$. Therefore, RC4 and RC6 contain the frequency feature out of the alpha band. So, the alpha rhythm cannot be effectively extracted due to the component mixing as a result of large embedding dimension.

**Figure 10:**
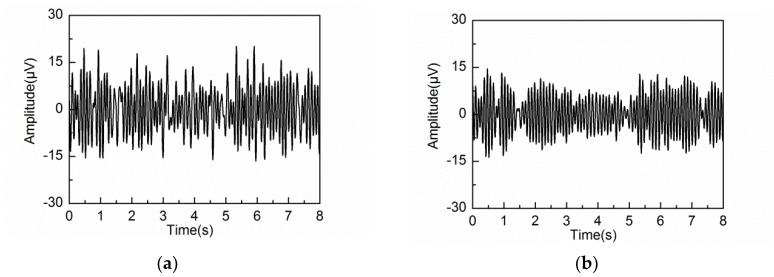

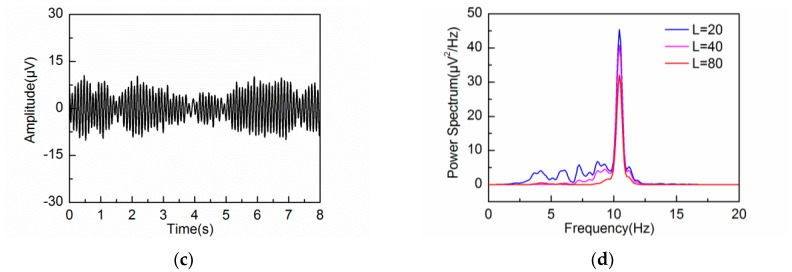
Extracted alpha rhythm under eyes-closed condition: (**a**) L = 20; (**b**) L = 40; (**c**) L = 80; (**d**) PSD of the extracted alpha rhythm by different L.

The reconstructed components containing the same frequency feature of the alpha rhythm are then grouped to calculate the alpha rhythm by the diagonal averaging step. The extracted alpha rhythms by three different embedding dimensions are shown in Figure 10a–c, while the PSD corresponding to the three embedding dimensions is shown in Figure 10d.

Figure 10d shows that strong alpha rhythms are extracted by L = 20 and 40, whose peak value of PSD are 45.40 and 40.87 ${\mu V}^{2}/\mathrm{Hz}$, respectively. However, the PSD by L = 20 contains large amounts of frequency components outside the alpha band. When the embedding dimension is set at L = 80, the PSD of the extracted alpha rhythm is within the alpha band well. However, the peak PSD value of the extracted alpha rhythm is 32.02 ${\mu V}^{2}/\mathrm{Hz}$, which is weaker than those by L = 20 and 40. This is caused by the problem of component mixing at a large embedding dimension. Some frequency components of the alpha rhythm are mixed together with the other reconstructed components.

**Figure 11:**
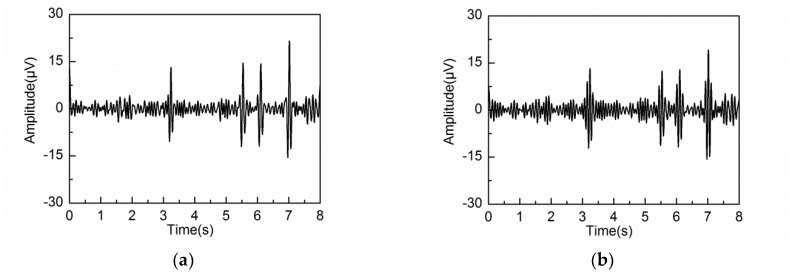

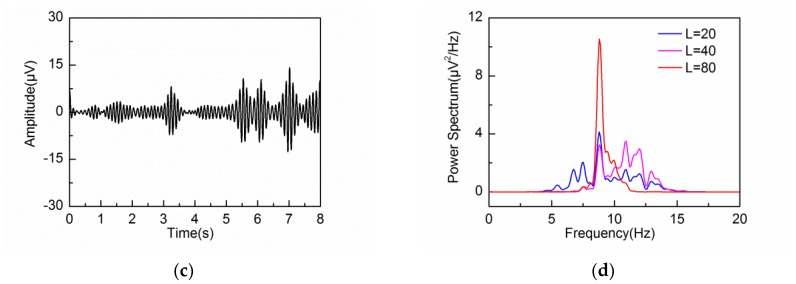
Extracted alpha rhythm under eyes-open condition: (**a**) L = 20; (**b**) L = 40; (**c**) L = 80; (**d**) PSD of the extracted alpha rhythm by different L.

**Figure 12:**
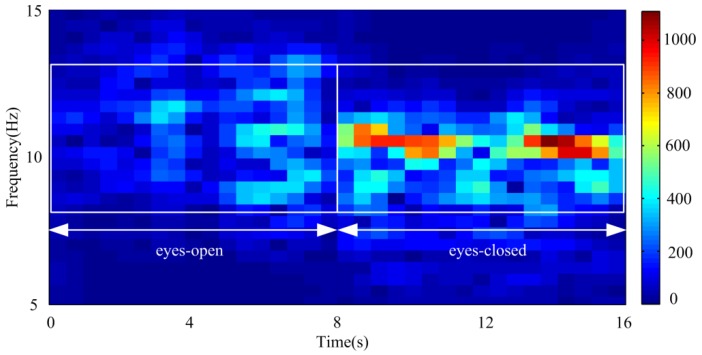
Spectrogram of the extracted alpha rhythm under eyes-open and eye-closed conditions by L = 40.

The extracted alpha rhythms and corresponding PSD by different embedding dimensions under eyes-open condition are shown in Figure 11. Prior research has demonstrated that the alpha rhythm in resting state with eyes closed is much stronger than that under eyes-open condition with visual stimulation [37]. It can be seen that the extracted alpha rhythm by L = 20 and 40 under eyes-open condition is much weaker than that under eyes-closed condition. But the extracted alpha rhythm by L = 80 under eyes-open condition has significant amplitude, which does not agree with reality. It is likely because some frequency components of the artifacts or noise are mixed into the alpha rhythm by a large embedding dimension. Based on the results analysis under both eyes-open and eyes-closed conditions, the extracted alpha rhythm by L = 40 shows better performance than those by L = 20 and 80. Figure 12 shows the spectrogram of the extracted alpha rhythm, which indicates the change process of power as a function of time and frequency. It presents significant difference between the eyes-open and eyes-closed states.

**Figure 13:**
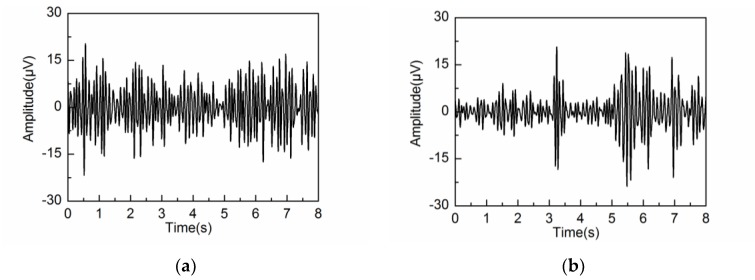
Extracted alpha rhythm by wavelet decomposition (WDec) under: (**a**) eyes-closed condition; (**b**) eyes-open condition.

**Figure 14:**
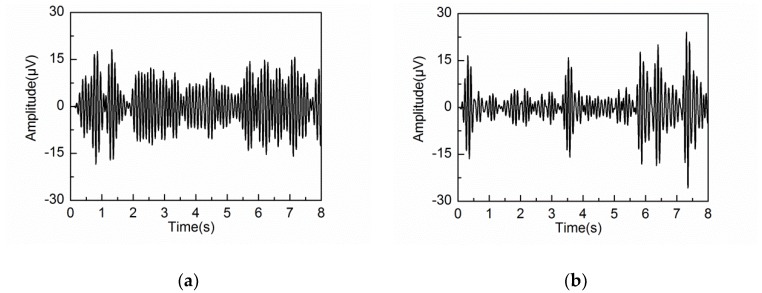
Extracted alpha rhythm by infinite impulse response (IIR) under: (**a**) eyes-closed condition; (**b**) eyes-open condition.

**Figure 15:**
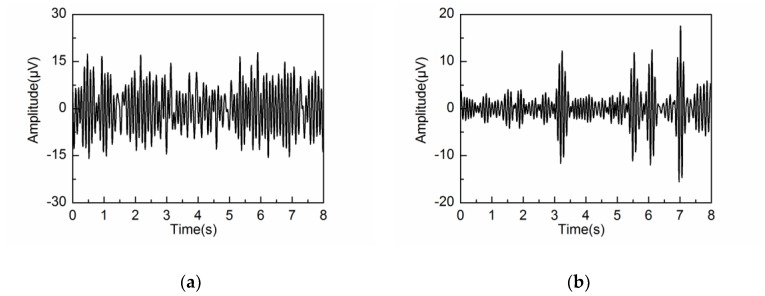
Extracted alpha rhythm by SSA# under: (**a**) eyes-closed condition; (**b**) eyes-open condition.

**Figure 16:**
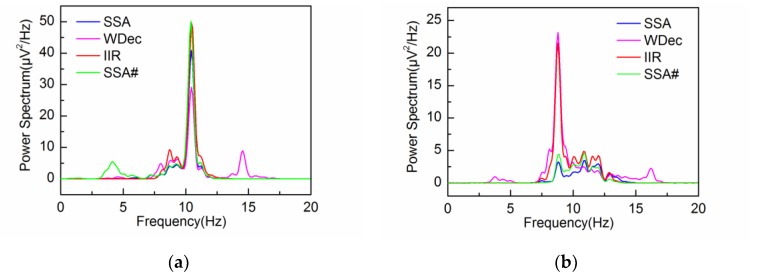
PSD of the extracted alpha rhythm by four methods: (**a**) under eyes-closed condition; (**b**) under eyes-open condition.

The performance of the adaptive SSA is compared with the other methods: wavelet decomposition (WDec), infinite impulse response (IIR) and another recently reported SSA method (SSA#) [22]. Figure 13, Figure 14 and Figure 15 show the extracted alpha rhythms under eyes-closed and eyes-open conditions using WDec, IIR and SSA#, respectively. For comparison, the PSD of the extracted alpha rhythms by four methods under eyes-closed and eyes-open conditions are shown in Figure 16.

As can be observed in Figure 16a, under eyes-closed condition, the adaptive SSA, IIR and SSA# can extract strong alpha rhythm. However, the extracted alpha rhythm by SSA# contains the frequency components out of the alpha band, which is due to the component mixing resulting from unsatisfied decomposition. In comparison, the extracted alpha rhythm by WDec is much weaker (29.18 ${\mu V}^{2}/\mathrm{Hz}$) with large amounts of frequency components falling outside the alpha band. Figure 16b shows that, under eyes-open condition, WDec can extract significant frequency components of alpha rhythm (23.17 ${\mu V}^{2}/\mathrm{Hz}$), which is close to that under eyes-closed condition. Clearly, it is inconsistent with reality. Because IIR is unable to remove artifacts and noise from the alpha rhythm with overlapping frequency spectrum, IIR yields a similar result of strong alpha rhythm. In the contrast, the extracted alpha rhythm by the adaptive SSA has low PSD amplitude, which can reflect the eyes-open state efficiently. As can be observed in Figure 16b, the extracted alpha rhythm by SSA# is similar to that by the adaptive SSA. In comprehensive consideration of the alpha rhythm extraction under eyes-open and eyes-closed conditions, the adaptive SSA performs better than WDec, IIR and SSA#.

In order to further verify the performance of the adaptive SSA, the extracted alpha rhythm is used to distinguish between eyes-open and eyes-closed states. At the same time, the classification result by the adaptive SSA is used to compare with those by WDec, IIR and SSA#, together with autoregressive model combined with linear discriminant analysis (AR) [38].

**Figure 17:**
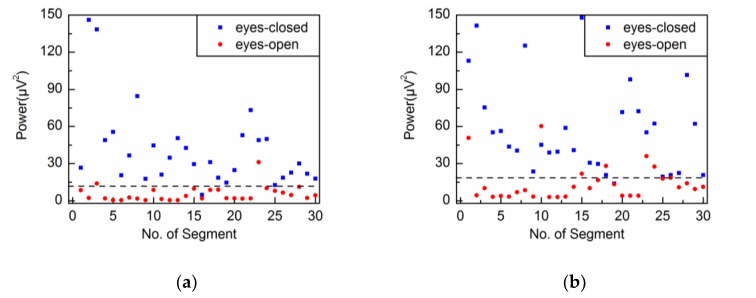

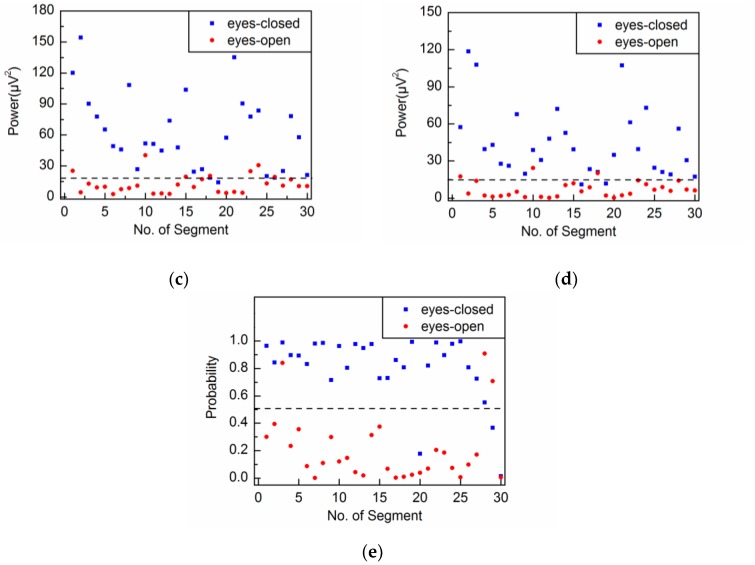
Classification results between eyes-closed and eyes-open states by: (**a**) adaptive SSA; (**b**) WDec; (**c**) IIR; (**d**) SSA#; (**e**) autoregressive model (AR).

In this study, the power ($P=\sum V_{\alpha }^{2}/N$) is selected as the characteristic parameter of the alpha rhythm [21], where $V_{\alpha }$ represents the amplitude of the extracted alpha rhythm. Figure 17a shows the power values of the extracted alpha rhythm by the adaptive SSA. Obviously, the power values under eyes-open condition are generally lower than those under eyes-closed condition. Then the threshold value is set at 12 ${\mu V}^{2}/\mathrm{Hz}$. When the power is higher than the threshold value, it is classified into the eyes-closed category. Otherwise, it is classified into the eyes-open category. Consequently, the accuracy of classification is 95.0%.

Figure 17b–d show the power values of the extracted alpha rhythms by WDec, IIR and SSA#, respectively. Similar to the results obtained by the adaptive SSA, the power values under eyes-open condition are generally lower than those under eyes-closed condition. With threshold values of 19 ${\mu V}^{2}/\mathrm{Hz}$, 18 ${\mu V}^{2}/\mathrm{Hz}$ and 15 ${\mu V}^{2}/\mathrm{Hz}$, WDec, IIR and SSA# achieve the best accuracy of classification: 88.3%, 86.7% and 91.7%, respectively. It can be seen that the threshold value by SSA# is a little greater than that by the adaptive SSA. It is likely because SSA# suffers from component mixing and therefore the extracted alpha rhythm contains the frequency components out of alpha band. The threshold values by WDec and IIR are much greater than that by the adaptive SSA. It may be because WDec and IIR cannot reduce artifacts and noise contamination that has the same frequency components as the alpha band. Figure 17e shows the classification result by AR and the threshold value is set at the probability of 0.5. When the probability is higher than the threshold value, it is classified into the eyes-closed category. Otherwise, it is classified into the eyes-open category. The accuracy of classification by AR is 90.0%. Therefore, the classification performance by the adaptive SSA is better than those by WDec, IIR, SSA# and AR. It is concluded that the adaptive SSA assisted by the embedding dimension selection method could be potentially used to distinguish between eyes-open and eyes-closed states.

## 5. Conclusions

In this study, a method for selecting the embedding dimension is proposed for extracting brain rhythm from EEG signal by adaptive SSA. Based on the embedding dimension selection method, the frequency bandwidth of each reconstructed component is constrained to a particular band of the brain rhythm of interest. At the same time, it avoids component mixing in the SVD step. Simulated EEG signal based on MPA EEG model and real EEG signal under eyes-open and eyes-closed conditions are used to verify the proposed method. Experimental results show that alpha rhythms can be effectively extracted by the adaptive SSA assisted by the embedding dimension selection method. The accuracy of classification between eyes-open and eyes-closed states is 95.0%, better than the corresponding values by WDec (88.3%), IIR (86.7%), SSA# (91.7%) and AR (90.0%).

## Figures and Tables

**Table 1:** Parameters of the simulated electroencephalography (EEG) signal.

	Symbol	Value	Comment
Spontaneous EEG	$f_{1}$/Hz	2.50	Delta rhythm
	$\gamma_{1}$	0.99	
	$\sigma_{1}^{\xi }$	2.26	
	$f_{2}$/Hz	6.00	Theta rhythm
	$\gamma_{2}$	0.97	
	$\sigma_{2}^{\xi }$	2.78	
	$f_{3}$/Hz	10.50	Alpha rhythm
	$\gamma_{3}$	0.99	
	$\sigma_{3}^{\xi }$	2.35	
	$f_{4}$/Hz	21.50	Beta rhythm
	$\gamma_{4}$	0.99	
	$\sigma_{4}^{\xi }$	0.36	
Artifacts	$V_{\mathrm{EOG}}$/μV	50	Amplitude of EOG
	$T_{\mathrm{EOG}}$/s	3	Period of EOG
	$W_{\mathrm{EOG}}$/s	0.3	Pulse width of EOG
	$V_{\mathrm{BD}}$/μV	10	Amplitude of baseline drift
	$f_{\mathrm{BD}}$/Hz	0.5	Frequency of baseline drift
Noise	$P_{n}$/dBW	1	Power of white noise
